# Exercise-Induced Extracellular Vesicles Delay the Progression of Prostate Cancer

**DOI:** 10.3389/fmolb.2021.784080

**Published:** 2022-01-11

**Authors:** Lilite Sadovska, Jānis Auders, Laura Keiša, Nadezhda Romanchikova, Laila Silamiķele, Madara Kreišmane, Pawel Zayakin, Satoru Takahashi, Zane Kalniņa, Aija Linē

**Affiliations:** ^1^ Cancer Biomarker Group, Latvian Biomedical Research and Study Centre, Riga, Latvia; ^2^ Faculty of Medicine, University of Latvia, Riga, Latvia; ^3^ Laboratory Animal Core Facility, Latvian Biomedical Research and Study Centre, Riga, Latvia; ^4^ Department of Experimental Pathology and Tumor Biology, Nagoya City University Graduate School of Medical Sciences, Nagoya, Japan; ^5^ Faculty of Biology, University of Latvia, Riga, Latvia

**Keywords:** extracellular vesicles, exercise, prostate cancer, RNA cargo, RNA sequencing

## Abstract

Increasing evidence suggests that regular physical exercise not only reduces the risk of cancer but also improves functional capacity, treatment efficacy and disease outcome in cancer patients. At least partially, these effects are mediated by the secretome of the tissues responding to exercise. The secreted molecules can be released in a carrier-free form or enclosed into extracellular vesicles (EVs). Several recent studies have shown that EVs are actively released into circulation during physical exercise. Here, we for the first time investigated the effects of exercise-induced EVs on the progression of cancer in an F344 rat model of metastatic prostate cancer. Although we did not observe a consistent increase in the circulating EV levels, RNA sequencing analysis demonstrated substantial changes in the RNA content of EVs collected before and immediately after forced wheel running exercise as well as differences between EVs from runners at resting state and sedentary rats. The major RNA biotype in EVs was mRNA, followed by miRNA and rRNA. Molecular functions of differentially expressed RNAs reflected various physiological processes including protein folding, metabolism and regulation of immune responses triggered by the exercise in the parental cells. Intravenous administration of exercise-induced EVs into F344 rats with orthotopically injected syngeneic prostate cancer cells PLS10, demonstrated reduction of the primary tumor volume by 35% and possibly—attenuation of lung metastases. Hence, our data provide the first evidence that exercise-induced EVs may modulate tumor physiology and delay the progression of cancer.

## Introduction

Prostate cancer (PC) is the second most frequently diagnosed cancer in males worldwide affecting more than 1.4 million men per year. In terms of mortality, PC is the fifth leading cause of death from cancer in men (Ferlay et al., 2015). Hence, PC is a global health problem requiring effective primary, secondary, and tertiary prevention measures. Regular physical activity is associated with a lower incidence of many common types of cancer (Moore et al., 2016), whereas the association between physical activity and PC risk remains controversial. Some studies have reported that leisure-time physical activity is associated with a higher risk of PC, some studies showed no clear relationship, however larger number of studies have found a decrease of PC incidence in physically active men and the effect showed a dose-response relationship with vigorous activity (Shephard, 2017). Furthermore, several studies have shown that exercise reduced fatigue and treatment side effects, improved quality of life, prevented disease recurrence, and improved survival of PC patients (Kenfield et al., 2011; Bourke et al., 2016; Shephard, 2017; Belloni et al., 2021). Moreover, experiments in murine tumor models have demonstrated that exercise leads to a significant reduction in tumor size and incidence, and these effects are associated with remodeling of the immune tumor microenvironment (TME) (Pedersen et al., 2016; Idorn and Thor Straten, 2017). This suggests that apart from the well-documented beneficial effects of exercise on cardiovascular fitness, energy balance and body weight, it may have a direct effect on cancer. Therefore, exercise may not just be preventive but also therapeutic and hence serve as an important tool for tertiary prevention of cancer.

At least partially, the effects of exercise are mediated by various molecules (proteins, lipids, RNAs, metabolites etc.) secreted into the circulation by muscle, bone, brain, liver and other tissues. These molecules can be secreted in a soluble form or packaged into carriers such as extracellular vesicles (EVs). The term “EVs” refers to all kinds of vesicles naturally released from cells that are delimited by a lipid bilayer and cannot replicate (Thery et al., 2018). According to the mode of biogenesis, three main types of EVs have been defined: exosomes, microvesicles and apoptotic bodies (Yanez-Mo et al., 2015). They differ in their molecular content, size, membrane composition, cellular source and specific functions. Although initially considered to be a waste disposal mechanism (Johnstone et al., 1987), it is now clear that EVs generated by both live and apoptotic cells interact with the recipient cells and are important mediators of intercellular communication (Tkach and Thery, 2016; Caruso and Poon, 2018). EVs can be internalized by the recipient cells and trigger various intracellular signal transduction pathways (Yanez-Mo et al., 2015; Zhan et al., 2021) or bind to the cell surface receptors and trigger the respective downstream signaling pathway (Muller et al., 2016; Muller et al., 2017).

Exercise has been shown to induce a rapid release of EVs into circulation (Fruhbeis et al., 2015; Whitham et al., 2018). A recent study demonstrated that exercise induced the secretion of over 300 proteins into EVs, including glycolytic enzymes and myokines, i.e. cytokines secreted by skeletal muscle (Whitham et al., 2018). Myokines have been shown to act as tumor suppressors and may impact several hallmark features of cancer (Hojman et al., 2011; Ruiz-Casado et al., 2017), whereas glycolytic enzymes might induce changes in the metabolic activity of cancer cells (Pedersen et al., 2015). We hypothesized that exercise-induced EVs may directly interact with cancer cells and alter their behavior as well as change the functional phenotype of circulating and tumor-infiltrating immune cells thus affecting the growth rate and metastatic potential of cancer. In this pilot study, we investigated the RNA content of EVs released during forced wheel running and their effects on the progression of cancer in a rat model of metastatic prostate cancer.

## Materials and Methods

### Animal Care and Experimental Design

The experimental procedures in animals were approved by the National animal welfare and ethics committee (permit no. 121/2021) and were performed in compliance with the Directive 2010/63/EU as adopted in the national legislation.

In total, 37 naïve SPF male Fischer 344 rats were obtained from Charles River Laboratories, Germany (immunocompetent inbred strain F344/DuCrl). During the introduction, animals were randomly allocated in cages in pairs or trios; individually ventilated cages GR900, HEPA-ventilated by SmartFlow air handling unit (Tecniplast, Italy) at 75 air changes per hour were used for animal housing. Access to autoclaved water acidified to pH 2.5–3.0 with HCl and standard rodent diet (4RF21 (A), Mucedola) was provided *ad libitum*. Aspen wooden bedding and nesting material (Tapvei, Estonia) together with rat cardboard houses (Velaz, Czech Republic) and aspen gnawing bricks (Tapvei, Estonia) were provided in all cages, and cages were changed every 7 days. Animals were housed in SPF facility under controlled temperature (24 ± 1°C) and relative humidity of 40–60%. Animal health monitoring was performed in line with FELASA recommendations (Mähler et al., 2014).

**FIGURE 1:**
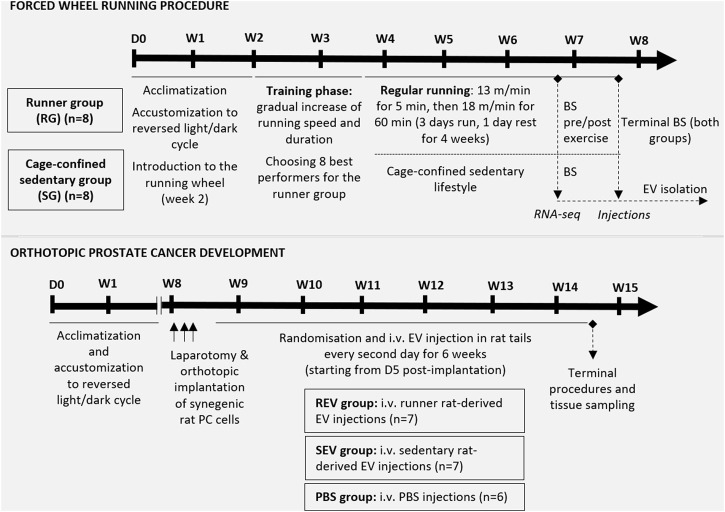
Schematic overview of the *in vivo* study design. The upper panel represents the steps of the forced wheel running procedure—in total, sixteen 7 w/o male F344 rats underwent a 2-weeks acclimatization period. During the second week, rats were introduced to the running wheel, which was followed by a 12-days training phase for all experimental animals. During the training phase, 8 best performers were chosen for the runner group while the remaining 8 rats were assigned to the cage-confined sedentary group. The running exercise phase lasted for 5 weeks in total, including 4 weeks of regular running in a setting described in the figure. After 4 weeks of exercise, blood samples were taken before and shortly after 1 h running from the runner group and a single blood sample from the control animals for further plasma EV analyses. At the endpoint, total blood sample was taken *via* cardiac puncture and used for plasma EV isolation. The lower panel shows the steps of *in vivo* evaluation of the effect of EVs (isolated from the endpoint plasma samples) on orthotopic PC development in syngeneic male F344 rats. Briefly, anesthetized 12 w/o male F344 rats were subjected to a laparotomic incision to enable orthotopic implantation of 5 × 10^6^ PLS10 rat prostate cancer cells into the ventral prostate. After stratified randomization, the animals were divided into 3 groups and, starting from day 5 post-implantation, rats from all 3 groups received i.v. injection of EVs or PBS as shown. After 6 weeks of injections, animals were sacrificed, and tissues of interest were collected. BS, blood sampling; D, day; i.v., intravenous; W, week.

All animals were subjected to at least 2-weeks acclimatization period with the adaptation to a reverse 12 h light/dark cycle (dark phase set to 10:00 am–10:00 pm; visible light intensity <25 lux), and then used to model either regular physical exercise (i.e. forced wheel running model) or orthotopic PC development [PLS10 rat PC model (Suzuki et al., 2015)]—see Figure 1 for schematic overview. An individual animal served as an experimental unit in both models. Before starting the procedures, the animals were identified by tattooing their tails using AIMS™ NEO-9 Neonate Tattooing System according to the manufacturer’s instructions.

### Cell Culture

Rat prostate cancer cell line PLS10 was developed previously using chemically-induced prostate carcinoma in F344 rats (Nakanishi et al., 1996; Suzuki et al., 2015). Cells were maintained in RPMI-1640 medium, supplemented with 10% FBS, 2 mM L-glutamine and 1x Antibiotic-Antimycotic at +37°C in a humidified atmosphere containing 5% CO2.

Cells in the exponential growth phase were trypsinized, counted and resuspended in PBS in aliquots of 5 × 10^6^ cells per 25 μL of PBS. The aliquots were immediately transferred to the animal facility and kept on ice water until injection. Just before injection into the rat prostate, the cell suspension was mixed with an equal volume of ice-cold Matrigel (Corning #356237, USA). Fresh cell suspensions were prepared for every batch of 3-4 animals undergoing laparotomic surgery.

### Forced Wheel Running Exercise

In total, 16 7-week-old male F344 rats were subjected to model regular exercise and sedentary lifestyle. The number of animals per group (*n* = 8) was chosen based on the available data on average EV plasma concentrations and calculations we made to ensure the necessary amount of EVs for the 6-weeks injection period in the syngeneic PC model animals (Figure 1). In the second week after arrival and acclimatization to the reversed light/dark cycle, rats were introduced to the forced running wheel (system for rats, model 80805A, Lafayette Instrument) without a specific running mode. It was followed by a 12-days training phase for all 16 experimental animals (started at 9 weeks of age) by gradually increasing the running speed and duration. During the training phase, 8 best performers were selected for the runner group while the remaining 8 rats were assigned to the sedentary group. After assigning to groups, the animals were kept co-housed with their initial cage mates.

The regular forced running exercise phase for the runner group lasted for 5 weeks in total, including 4 weeks of regular running in a setting described in Figure 1. After 4 weeks of regular exercise, rats underwent brief anesthesia with 1.5% isoflurane and 500 μL of blood from the tail vein were taken. This step was repeated for the same animals immediately after 1 h of running. On the same day, a blood sample was collected from the sedentary rats following the same protocol. After 5 weeks of regular exercise, all the animals immediately after 1 h running were subjected to deep surgical anesthesia with 5% isoflurane and underwent terminal blood collection *via* cardiac puncture. Blood was immediately collected into S-Monovette Hematology EDTA K3 tubes (Sarstedt, Germany) or BD Vacutainer™ Blood Collection Tubes with K2EDTA (Fisher Scientific, USA), depending on the volume, and centrifuged at 2000 x g, +4°C for 15 min. Plasma was collected in fresh tubes and immediately frozen for further EV isolation.

### Isolation of EVs

EVs were isolated from rat plasma by size exclusion chromatography (SEC) using qEVoriginal/35 nm or qEV10/35 nm columns (IZON, USA) depending on the plasma volume. The SEC fractions were analyzed using ZetaSizer Nano ZS (Malvern Panalytical, UK) and the fractions containing EVs were concentrated using Amicon Ultra-0.5, Ultracel-3 Membrane, 3 kDa centrifugal filter units (Merck Millipore, Germany). EVs that were used for RNA sequencing were treated with proteinase K (Thermo Fisher Scientific, USA) and RNAse A (Thermo Fisher Scientific, USA) to remove all the free proteins and RNAs that are not enclosed in EVs. EVs were visualized by transmission electron microscopy (TEM) and the size distribution profile and concentration of EVs were determined by nanoparticle tracking analysis (NTA) using NanoSight NS500 instrument (Malvern, UK) as described before (Endzelins et al., 2017).

### RNA Extraction

RNA was extracted from plasma EVs using miRNeasy micro kit (Qiagen, USA) according to the manufacturer’s protocol. The concentration and quality of the obtained RNA were analyzed on Agilent Bioanalyzer with Agilent RNA 6000 Pico chip (Agilent Technologies, Germany).

### RNA Sequencing and Data Analysis

RNA libraries were constructed using CleanTag® Small RNA Library Prep Kit (Trilink Biotechnologies, USA), the quality and concentration of obtained libraries were analyzed on Agilent Bioanalyzer using Agilent High Sensitivity DNA chip (Agilent Technologies, Germany). The libraries were cleaned using Blue Pippin DNA Size Selection with 3% gel Blue Pippin Cassette (Sage Science, USA) setting tight target length to 140 bp thus selecting fragments with size in tight range to 140 bp (126–154 bp). The libraries were diluted as required and sequenced on Illumina NextSeq500 instrument using NextSeq 500/550 Mid Output Kit v2.5 (150 cycles) (Illumina, USA).

The obtained raw data in fastq format were analyzed using *ad-hoc* R script pipeline, which included: adapter trimming [cutadapt (Martin, 2011)], read mapping [bowtie2 (Langmead and Salzberg, 2012)] against RGSC rat (*Rattus norvegicus*) genome (version Rnor_6.0), multi-aligned reads reposition [ShortStack (Axtell, 2013)], counting [Rsubread package (Liao et al., 2019)] with RGSC (version Rnor_6.0) and miRbase (Kozomara et al., 2019) annotations. For differentially expressed gene (DEG) analysis, the reads were normalized per sample, the reads mapped to features were counted and analyzed using quasi-likelihood F-tests by edgeR (McCarthy et al., 2012) package. A subset of DEGs (adj. *p* < 0.05) was subjected to GO terms [GOstats (Falcon and Gentleman, 2007)] and enrichment analyses (rentrez (Winter 2017), GO.db (Carlson, 2019a), org.Rn.eg.db (Carlson, 2019b) packages).

### Syngeneic Orthotopic Prostate Cancer Model

For modeling an orthotopic PC development, 21 F344 rats were used—the sample size of 7 animals per group (see Figure 1 for study groups) was calculated by statistical power analyses using G*Power software (Buchner et al., 2021) and taking into account 80% power and *α* = 0.05, the published tumor size variation (Suzuki et al., 2015), expected effect size and the chosen statistical test for the result analyses.

At the age of 12 weeks, each animal underwent laparotomic surgery by using an aseptic technique under 2.5% isoflurane anesthesia, and 5 × 10^6^ syngeneic rat prostate cells PLS10 in 50 μL total volume containing 50% Matrigel were injected into the ventral lobe of the prostate. The incisions were closed with Novosyn 4/0 DS19 mid-term absorbable sutures (B.Braun, Germany) and secured with surgical adhesive. An ophthalmic gel was provided during surgery and 8 ml of warm saline was administered subcutaneously immediately after surgery. Each animal received subcutaneous Meloxicam (1 mg/kg) injection during surgery and for 3 days post-surgery. Animals were single housed in sterile cages for 3 days and then returned to their home cages. Animals were closely monitored for possible postsurgical complications; the wounds were treated with furasol solution. In total, 7 rats divided in 2 sets were implanted with PC cells per day, and animals were allocated to the study groups after stratified randomization by considering this setting (Figure 1; one animal did not survive the surgery).

Starting from day 5 post-implantation, rats from all study groups *via* lateral tail vein received i.v. injections of 100 μL EV solution containing 1.5 × 10^10^ EVs in PBS or PBS every second day as shown in Figure 1. During PC development, animals were carefully monitored for possible signs of suffering (following the IACUC Policy #012), and the tumor size was estimated by palpation. After 6 weeks of EV injection, all study animals were humanely euthanized, and terminal blood samples and tissues of interest were collected and fixed in 10% buffered formalin. Primary tumors were measured, and tumor volume was calculated using the following formula: tumor volume = (width)2 x length/2.

### Statistical Analysis

A nonparametric one-tailed Mann-Whitney test was used to compare the tumor volume and the number of metastases between groups of animals. Fisher’s exact test was used to compare the number of animals with/without metastases between groups of animals. *p*-value of ≤0.05 was considered to be significant. The statistical analyses were performed with GraphPadPrism 7 (GraphPad, USA).

## Results

### Effect of Forced Wheel Running Exercise on the Plasma EV Levels

**FIGURE 2:**
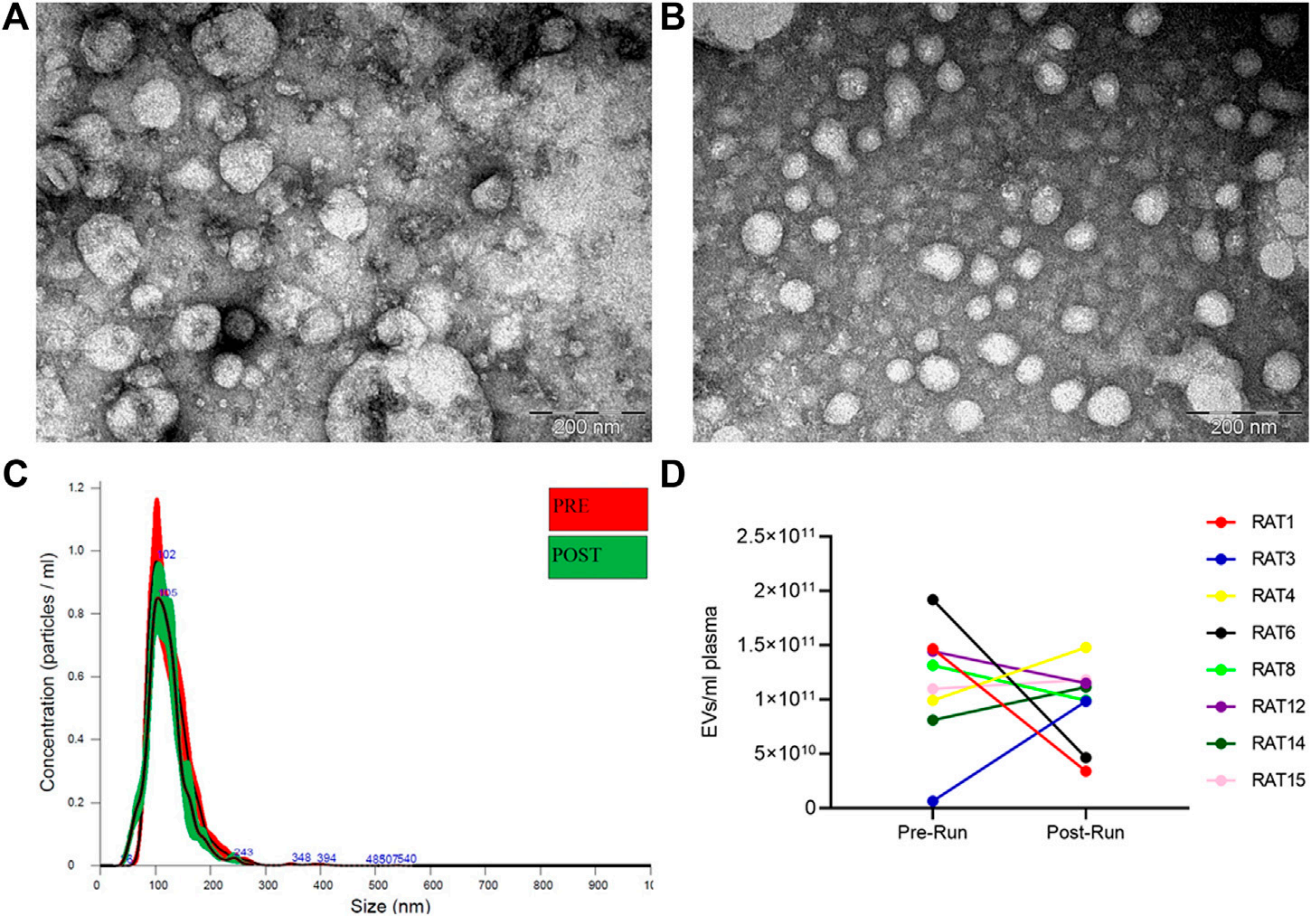
Characteristics of EVs released in plasma during forced wheel running exercise. **(A)** Representative TEM image of Pre-RUN plasma EVs. **(B)** Representative TEM image of Post-RUN plasma EVs. **(C)** Quantity and size distribution of Pre-RUN and Post-RUN plasma EVs assessed by NTA (a representative case). **(D)** Paired dot plots show the number of particles in Pre-RUN and corresponding Post-RUN plasma samples determined by NTA.

EVs were isolated from rat plasma before (Pre-RUN) and immediately after forced wheel-running exercise (Post-RUN) and the yield, size and purity of EVs were assessed by TEM and NTA. TEM images revealed that the majority of particles were ranging from 30 to 160 nm in diameter and had a cup-shaped morphology that is typically observed for exosomes in TEM. However, smaller particles that possibly represent lipoprotein particles and a small number of large particles of 200–250 nm in diameter were also present in the majority of the samples analyzed and we did not observe any significant differences between Pre-RUN and Post-RUN samples (Figure 2A,B). NTA showed that the major fraction of particles was in the size range from 36 to 200 nm and the concentrations of EVs ranged from 6.5 × 10^9^ to 1.9 × 10^11^ particles per ml of plasma (Figure 2C). However, no significant differences neither in size or concentration of EVs between Pre-RUN and Post-RUN samples were observed (Figure 2D).

### Changes in the EV RNA Content During Exercise

To determine whether exercise affects the EV-enclosed RNA cargo, we performed RNA sequencing analysis of Pre-RUN (*n* = 8) and Post-RUN (*n* = 8) plasma EVs from the runner group and plasma EVs from the sedentary group (*n* = 8). Analysis of the total EV RNA by Bioanalyzer showed the dominant peaks in the range from 20 to 150 nt (data not shown). The total EV RNA was used for the RNA-seq library construction without a prior size selection. Importantly, prior to the RNA extraction, EVs were treated with Proteinase K and RNase A to remove RNAs that are attached to the surface of EVs. On average, 5.9 million raw reads were obtained per library, however, on average 2.85 million reads per library remained after the quality control, adapter trimming, and filtering out the reads that were shorter than 16 nt. The average overall alignment rate to the *Rattus norvegicus* genome was 51.5%. The aligned reads were counted using Rsubread package (Liao et al., 2019) and RGSC rat genome and genome annotation (version Rnor_6.0). Results showed that the majority of the reads were mapped to mRNAs (73.8%), followed by miRNAs (13.6%) and rRNAs (6.7%), while the other biotypes—lincRNAs, mitochondrial rRNAs, processed pseudogenes etc. constituted less than 2% each. However, when the reads were counted using miRBase annotation, 0.6% of the mapped reads were counted as mature miRNAs and a total of 194 different miRNAs were identified.

**FIGURE 3:**
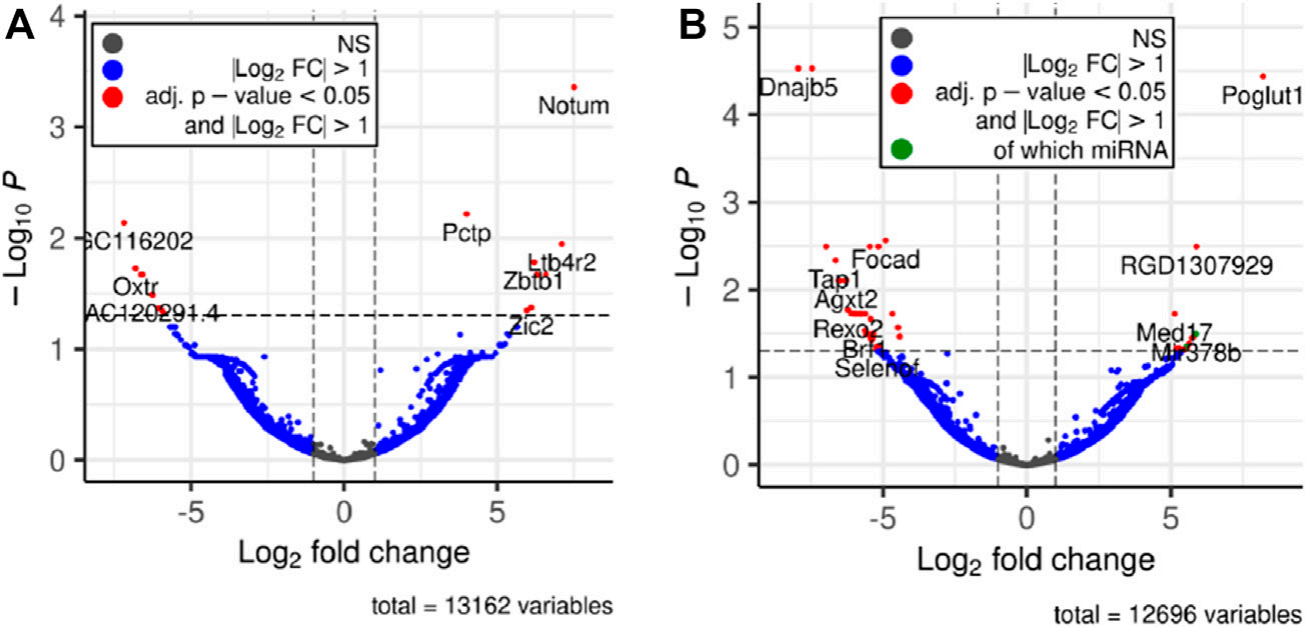
Volcano plots depicting significant changes in the EV-enclosed RNA content. **(A)** Post-RUN *vs.* Pre-RUN EVs from exercised rats. **(B)** Pre-RUN EVs from exercised rats *vs.* EVs from sedentary control group rats.

Next, we performed differential expression analysis using edgeR package. RNAs which expression was detected in less than 4 samples were excluded from the analysis. To assess whether the RNA cargo of EVs that are released into the circulation during the forced wheel running exercise is distinct from that in the resting state, Post-RUN EVs were compared against the Pre-RUN EVs. A total of 20 differentially expressed genes (DEGs) (adj. *p* < 0.05)—10 upregulated and 10 downregulated during the exercise were identified (Figure 3A; Supplementary Table S1). The top 10 DEGs are shown in Table 1. All DEGs are protein-coding genes and no differentially expressed RNAs were found in other RNA biotypes. GO term enrichment analysis of DEGs revealed enrichment of genes related to unfolded protein binding (adj. *p* = 0.048).

**TABLE 1:** Top 10 differentially expressed genes in Post-RUN *vs.* Pre-RUN EVs.

Gene name	Description	Function Smith et al. (2020)	Expression Yu et al. (2014)	LogFC	Adjusted *p*-value
Notum	NOTUM, palmitoleoyl-protein carboxylesterase	Regulation of Insulin-like Growth Factor (IGF) transport and uptake by Insulin-like Growth Factor Binding Proteins (IGFBPs)	Selective expression in Liver (RPKM 96.2), Thymus (RPKM 23.3) and 3 other tissues	7.51	0.0004
Pctp	Phosphatidylcholine transfer protein	Plays a role in intermembrane transfer of phosphatidylcholines	Selective expression in Liver (RPKM 243.0), Testes (RPKM 114.6) and 8 other tissues	4.00	0.0061
MGC116202	LOC688736 uncharacterized protein KIAA0895-like	—	Selective expression in Brain (RPKM 59.9), Testes (RPKM 34.1) and 6 other tissues	−7.19	0.0073
Ltb4r2	Leukotriene B4 receptor 2	Mouse homolog is a chemoattractant for myeloid leukocytes	Selective expression in Lung (RPKM 1.6), Thymus (RPKM 1.1) and 9 other tissues	7.11	0.0113
Zbtb1	Zinc finger and BTB domain containing 1	May bind DNA	Selective expression in Thymus (RPKM 102.4), Spleen (RPKM 62.9) and 9 other tissues	6.21	0.0166
Oxtr	Oxytocin receptor	G-protein coupled receptor for the peptide hormone, oxytocin	Selective expression in Adrenal (RPKM 17.2), Uterus (RPKM 11.7) and 7 other tissues	−6.81	0.0188
Dnajb5	DnaJ (Hsp40) homolog, subfamily B, member 5	Predicted to be involved in chaperone cofactor-dependent protein refolding and response to unfolded protein	Selective expression in Muscle (RPKM 168.1), Brain (RPKM 82.7) and 9 other tissues	6.28	0.0213
Hspa5	Heat shock protein family A (Hsp70) member 5	secreted protein of the endoplasmic reticulum; may be involved in the assembly of secreted and membrane-bound proteins	Selective expression in Liver (RPKM 2693.8), Heart (RPKM 2015.3) and 9 other tissues	−6.64	0.0213
Alox5	Arachidonate 5-lipoxygenase	catalyzes the conversion of arachidonate to leukotriene A4 in leukotriene metabolism	Selective expression in Lung (RPKM 53.5), Heart (RPKM 25.7) and 9 other tissues	−6.57	0.0213
Dxo	Decapping exoribonuclease	Hydrolyzes the nicotinamide adenine dinucleotide (NAD) cap from a subset of RNAs	Selective expression in Adrenal (RPKM 116.7), Kidney (RPKM 46.2) and 9 other tissues	6.59	0.0213

Abbreviations: RPKM, reads per kilobase per million mapped reads (mean values given).

To assess the long-term effects of exercise on the RNA cargo of circulating EVs, Pre-RUN EVs were compared to the plasma EVs from the sedentary control rats. A total of 52 genes were differentially expressed (adj. *p* < 0.05), including 50 protein-coding genes and 2 miRNAs (Figure 3B; Supplementary Table S2). Only eleven of these RNAs had higher levels in the Pre-RUN EVs than in sedentary control EVs. The top 10 DEGs are shown in Table 2. GO term enrichment analysis showed the enrichment of genes associated with molecular functions “selenium binding” and “oxidoreductase activity, acting on peroxide as acceptor” (both adj, *p* = 0.025).

**TABLE 2:** Top 10 differentially expressed genes in Pre-RUN *vs*. sedentary control EVs.

Gene name	Description	Function Smith et al. (2020)	Expression Yu et al. (2014)	LogFC	Adjusted *p*-value
Dnajb5	DnaJ heat shock protein family (Hsp40) member B5	Predicted to be involved in chaperone cofactor-dependent protein refolding and response to unfolded protein	Selective expression in Muscle (RPKM 168.1), Brain (RPKM 82.7) and 9 other tissues	−7.95	0.00003
Ripor3	RIPOR family member 3	Orthologous to human RIPOR3, enables protein binding	Selective expression in Kidney (RPKM 21.2), Liver (RPKM 17.8) and 9 other tissues	−7.46	0.00003
Poglut1	Protein O-glucosyltransferase 1	Involved in the pathway protein glycosylation	Selective expression in Thymus (RPKM 128.2), Brain (RPKM 117.2) and 9 other tissues	8.21	0.00004
Focad	Focadhesin	Predicted to be located in focal adhesion. Orthologous to human FOCAD	Selective expression in Brain (RPKM 101.0), Muscle (RPKM 96.0) and 9 other tissues	−4.91	0.0027
Gpx3	Glutathione peroxidase 3	Catalyze the reduction of organic hydroperoxides and hydrogen peroxide (H2O2) by glutathione, and thereby protect cells against oxidative damage	Expression restricted to heart (RPKM 20.0), kidney (RPKM 587.3), lung (RPKM 25.6)	−6.97	0.0032
AC107331.1	—	Predicted to enable G protein-coupled receptor activity and olfactory receptor activity	—	−5.16	0.0032
RGD1307929	Similar to CG14967-PA	Orthologous to human KIAA0100, May be involved in membrane trafficking	Selective expression in Kidney (RPKM 513.2), Adrenal (RPKM 381.1) and 9 other tissues	5.89	0.0032
Lpin2	Lipin 2	Predicted to be involved in several processes, including cellular response to insulin stimulus; fatty acid catabolic process; and triglyceride biosynthetic process	Selective expression in Kidney (RPKM 124.7), Spleen (RPKM 111.6) and 9 other tissues	−5.46	0.0032
Tap1	Transporter 1, ATP binding cassette subfamily B member	May transport antigenic peptides across the endoplasmic reticulum membrane in preparation for MHC class I presentation	Selective expression in Thymus (RPKM 337.5), Spleen (RPKM 282.5) and 9 other tissues	−6.65	0.0046
Agxt2	Alanine-glyoxylate aminotransferase 2	Predicted to enable alanine-glyoxylate transaminase activity and beta-alanine-pyruvate transaminase activity	Expression restricted to liver (RPKM 29.2) and kidney (RPKM 110.3)	−6.28	0.0078

Abbreviations: RPKM, reads per kilobase per million mapped reads (mean values given).

### Effect of Exercise-Induced EVs on the Progression of Prostate Cancer

Next, we investigated the effects of EVs released during the forced wheel running exercise on the progression of *p*C. Briefly, total blood plasma EVs were isolated from F344 rats subjected to forced running wheel exercise or sedentary lifestyle, and the obtained EVs were subsequently administered intravenously in F344 rats with orthotopically injected syngeneic prostate cancer cells PLS10 (established from chemically induced, castration-resistant metastatic PC). The PLS10 PC rat tumor model was adopted based on the protocol originally published by the authors (Suzuki et al., 2015). PC-bearing rats received EV injections during 6 weeks of PC progression (representing stage 1 to stage 3/4 PC development)—REV group received Post-RUN EVs from runners, SEV group—EVs from sedentary rats, PBS group—vehicle only (Figure 1).

Six weeks after implantation, tumor masses were found in the ventral prostate of all rats. Noteworthy, no suffering was observed till the endpoint for the majority of tumor-bearing animals. However, two rats from each group were humanely euthanized before the set endpoint of 6 weeks due to signs of suffering. Postmortem analyses revealed that all the animals had tumor masses developed outside the original injection site leading to the stricture of the urethra, a common complication seen in orthotopic rodent PC models [our unpublished data (Suzuki et al., 2015)]. These animals were excluded from the data analyses.

**FIGURE 4:**
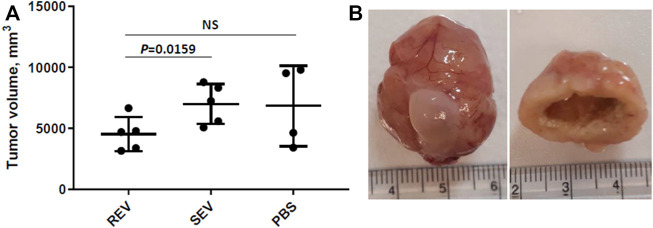
Effects of exercise-induced EVs on the progression of prostate cancer. **(A)** Comparison of primary tumor volumes between groups after 6 weeks of EV or vehicle administration. In the dot plot, mean tumor volumes are shown, whiskers represent standard deviation; dots represent individual animals. Mann-Whitney test was performed to assess the statistical significance of the differences between groups. **(B)** A representative example of an orthotopic PLS10 cell-induced prostate tumor (with bladder) and necrosis found in the center of most tumors.

Primary tumor volume was assessed as the primary outcome measure and compared between the groups. The mean primary tumor volumes were 4532 ± 1396 mm^3^ in the REV group, 7000 ± 1882 mm^3^ in SEV group, and 6841 ± 3291 mm^3^ in PBS group (Figure 4A). Statistically significant differences were seen only when comparing mean tumor volume of rats that received runner-derived EVs and those of SEV group animals (reduction of the primary tumor volume in REV group by 35%, Mann-Whitney *p* = 0.0159), but the difference was insignificant when compared to the PBS group, due to the small number of animals and large variation in tumor size in this group. All the primary tumors were found to have profound necrosis in the center of the tumor (Figure 4B).

Apart from tumor volumes, the location and number of macroscopic metastatic lesions were assessed. The results were highly inconsistent between individual animals and groups—metastases were found in various locations, including seminal vesicles, mesenteric, iliac and intestinal lymph nodes, and lungs (Table 3) and revealed no statistically significant differences between any of the study groups in terms of numbers or locations. However, distal lung metastases were observed only in the animals from control groups, but not rats receiving Post-RUN EVs (REV group) implying for a possible protective role of the EVs in cancer progression.

**TABLE 3:** Overview of the detected macrometastases in F344 PC model rats.

Group	Total No of metastatic lesions	No of rats with metastases/No of rats per group	Metastatic locations^a^					
			Lung	Peritoneal	Mesenterial	Seminal vesicle	Pancreatic	Intestinal
**REV**	4	3/5	0/0	1/1	1/1	0/0	0/0	2/2
**SEV**	8	3/5	2/2	2/1	0/0	0/0	0/0	4/2
**PBS**	18	3/4	11/2	3/2	1/1	2/1	1/1	0/0

a The number of total metastatic lesions in the given location/the number of animals with metastases in the given location.

## Discussion

As several previous studies had demonstrated that exercise increases the levels of circulating EVs both in human subjects and laboratory animals (Fruhbeis et al., 2015; Bei et al., 2017; Oliveira et al., 2018; Whitham et al., 2018; Neuberger et al., 2021), we assessed the EV concentration in rat plasma samples collected before and immediately after forced 1-h wheel running exercise. Although the EV levels were increased in some animals, the mean EV concentration and size were not significantly different in the Pre-RUN and Post-RUN plasma samples. These results are in line with several other studies finding no significant changes in the EV concentration after the exercise (Lovett et al., 2018; Hou et al., 2019; Yoon et al., 2021). This controversy suggests that different types, intensity and duration of exercise may affect the kinetics of EV release from various cell types or their clearance from the circulation in different ways. It could be possible that the induction of EV release during wheel running exercise is too slow to observe a substantial increase after 1 h or is compensated by increased clearance rate. Such a version is supported by Fruhbeis et al. showing that small EV levels increased immediately after cycling exercise and declined within 90 min at rest, whereas the increase was moderate but more sustained in response to treadmill running (Fruhbeis et al., 2015). Alternatively, these results may be affected by the use of different EV isolation and quantification methods and the variability of these methods may obscure a moderate increase in EV levels. In this study, we used IZON qEVoriginal/35 nm columns that have an optimum recovery range of 35–350 nm, thus larger microvesicles may be underrepresented in our EV preps, whereas co-isolation of larger lipoprotein particles is possible. Indeed, TEM images revealed small but consistent co-isolation of lipoprotein particles and the presence of some large particles that may represent lipoprotein aggregates, which may affect the NTA measurements. Hence, immunoisolation of EV subpopulations using EV surface markers may give a better insight into the dynamics of EV release and the cell types contributing to the pool of circulating EVs (Brahmer et al., 2019; Rigamonti et al., 2020).

Despite the fact that we did not observe a consistent increase of the circulating EV levels during exercise, RNA sequencing analysis revealed substantial differences in the RNA cargo of Pre-RUN and Post-RUN EVs from exercised rats, thus suggesting that EVs were actively released in the circulation in response to the forced wheel running exercise. Although selective RNA sorting mechanisms have been described that bias the RNA profiles of EVs (O'Brien et al., 2020), at least partially the molecular composition of EVs reflect that of the parental cell and therefore the analysis of RNA cargo may reveal the cell types producing EVs during the exercise. All of the 20 DEGs altered in the Post-RUN EVs were protein-coding genes. According to rat transcriptomic BodyMap (Yu et al., 2014), most of the genes that are upregulated in the Post-RUN EVs have tissue-selective or restricted expression pattern with the highest expression levels in the liver, testis, lung, muscle, brain, thymus and kidney. At least in human blood, the major sources of cell-free RNAs are blood cells, followed by much smaller fractions derived from spleen, liver and other tissues (Larson et al., 2021). Our data suggest that fractions of cell-free RNAs derived from organs involved in exercise are increased during the exercise relatively to the blood cell-derived fraction.

The differentially expressed genes in the Post-RUN EVs reflect the physiological processes that are triggered in various cell types in response to exercise. For example, 3 DEGs—*Notum* (palmitoleoyl-protein carboxylesterase), *Pctp* (phosphatidylcholine transfer protein) and *Cyp4b1* (cytochrome P450, family 4, subfamily b, polypeptide 1), which are induced in Post-RUN EVs, are implicated in various metabolic processes. Two other DEGs—*Dnajb5* (DnaJ heat shock protein family (Hsp40) member B5) and *Hspa5* (heat shock protein family A (Hsp70) member 5) are molecular chaperones that are involved in maturation, re-folding and degradation of proteins and play pivotal roles in cell survival under various stress conditions (Archer et al., 2018). Another set of genes is involved in the regulation of immune responses. *Ltb4r2* (leukotriene B4 receptor 2) and *Alox5* (arachidonate 5-lipoxygenase) are involved in leukotriene signaling that play important role in acute and chronic inflammation (Jo-Watanabe et al., 2019), while *Zbtb1* (zinc finger and BTB domain containing 1) is a transcription factor that is essential for the development, differentiation and effector function of T cells (Cheng et al., 2021). *Fcrlb* (Fc receptor-like B) is an F_C_ receptor homolog expressed as an intracellular protein in germinal center B cells (Wilson and Colonna, 2005).

We did not find significantly altered miRNAs, when compared the Post-RUN EVs with the Pre-RUN EVs. On the contrary, a recent study by Oliveira et al. found 12 miRNAs that were differentially expressed in rat serum EVs in response to treadmill running (Oliveira et al., 2018). The levels of eight of these miRNAs (rno-miR-128-3p, 103-3p, 148a-3p, 191a-5p, 10b-5p, 93-5p, 25-3p and 142-5p) were also altered in the Post-RUN EVs in our study, however, the difference did not reach the statistical significance.

Comparison of Pre-RUN EVs from exercised rats with EVs from sedentary control rats revealed 52 DEGs thus showing that exercise has a long-lasting effect on the circulating EV RNA cargo. Among the genes downregulated in the EVs from exercised rats were *Gpx3* (glutathione peroxidase 3), *Pxdn* (peroxidasin) and *Selenof* (selenoprotein F) that are associated with the molecular function “oxidoreductase activity, acting on peroxide as acceptor”; two of them—*Gpx3* and *Selenof* are also associated with “selenium binding”. Although several studies have shown that the levels of oxidative stress markers such as glutathione peroxidase increase during the exercise (Khcharem et al., 2021; Mesquita et al., 2021), our results suggest that their baseline levels are lower in exercised than in sedentary animals. Among the DEGs were two miRNAs—miR378b and miR35, that were present in the runner EVs but undetectable in the sedentary rat EVs. Both of them are implicated in the regulation of various processes in the body, including insulin sensitivity (Li et al., 2020) and protection against insulin resistance (Chen et al., 2019).

Several previous studies have investigated the effects of exercise on tumor incidence and progression in animal models. Nilsson et al. studied the effects of early-onset, lifelong voluntary wheel running in a naturally aging mouse model and showed that exercise protected against multiple types of cancer, lowered systemic inflammation and extended the health-span of naturally aged mice (Nilsson et al., 2019). Another study showed that voluntary wheel running reduced tumor incidence and growth across 5 different tumor models, including subcutaneous injection of B16F10 murine melanoma cells, intravenous injection of B16F10 melanoma cells, diethylnitrosamine (DEN)—induced liver cancer, Lewis Lung carcinoma model, and Tg(Grm1)EPv transgenic male mice model. In the subcutaneous B16 models, tumors from running mice showed higher infiltration of NK cells, CD3^+^ T cells and dendritic cells, while the metastasis model had infiltrated NK cells only (Pedersen et al., 2016), suggesting that the mobilization or increased cytotoxicity of NK cells may be one of the mechanisms of action.

Here, we for the first time studied the effects of exercise-induced EVs on the progression of cancer in a rat model of metastatic prostate cancer. Results showed that regular injections of exercise-induced EVs into tumor-bearing rats reduced the primary tumor growth by ∼35% and possibly may have delayed the development of lung metastasis. Pedersen et al. proposed four key mechanisms implicated in exercise-mediated cancer protection: normalization of tumor vasculature and blood flow, boosting immune cell functions, reprogramming metabolic pathways in cancer cells and controlling tumor growth by bioactive molecules secreted by skeletal muscle during contraction (Pedersen et al., 2015). In principle, exercise-induced EVs may contribute to all of these mechanisms, however, what is the scale of their contribution and which of the mechanisms dominate in the exercise-induced EV-mediated cancer protection remains to be established.

A shortcoming of our study, however, is the small sample size that remained after excluding animals showing the signs of suffering and large variation of the obtained data, therefore the results of this exploratory study should be regarded as indicative and should be validated in a larger cohort of animals. Moreover, the biodistribution, uptake and intracellular fate of the exercise-induced EVs and the effects triggered in the recipient cells remains to be investigated.

In summary, we show that the RNA cargo of EVs released into the circulation during exercise is altered as compared to the resting state and provide evidence that exercise-induced EVs may modulate tumor physiology and delay the progression of cancer thus supporting the idea that regular physical exercise should be prescribed to prostate cancer patients as a tertiary prevention measure.

## Data Availability

The datasets presented in this study can be found in online repositories. The names of the repository/repositories and accession number(s) can be found below: https://www.ebi.ac.uk/arrayexpress/, E-MTAB-11020.
